# Phylogenomic analysis of 343 *Xanthomonas citri* pv. citri strains unravels introduction history and dispersal paths

**DOI:** 10.1371/journal.ppat.1011876

**Published:** 2023-12-15

**Authors:** Jin Xu, Yanan Zhang, Jinyun Li, Doron Teper, Xiaoan Sun, Debra Jones, Yayu Wang, Jin Tao, Erica M. Goss, Jeffrey B. Jones, Nian Wang

**Affiliations:** 1 Citrus Research and Education Center, Department of Microbiology and Cell Science, IFAS, University of Florida, Lake Alfred, Florida, United States of America; 2 Florida Department of Agriculture and Consumer Services, Gainesville, Florida, United States of America; 3 State Key Laboratory of Agricultural Genomics, BGI-Shenzhen, Shenzhen, China; 4 Guangdong Magigene Biotechnology Co., Ltd., Guangzhou, China; 5 Department of Plant Pathology, IFAS, University of Florida, Gainesville, Florida, United States of America; 6 Emerging Pathogens Institute, University of Florida, Gainesville, Florida, United States of America; Sainsbury Laboratory, UNITED KINGDOM

## Abstract

*Xanthomonas citri* pv. *citri* (Xcc) causes the devastating citrus canker disease. Xcc is known to have been introduced into Florida, USA in at least three different events in 1915, 1986 and 1995 with the first two claimed to be eradicated. It was questioned whether the Xcc introduction in 1986 has been successfully eradicated. Furthermore, it is unknown how Xcc has spread throughout the citrus groves in Florida. In this study, we investigated the population structure of Xcc to address these questions. We sequenced the whole genome of 343 Xcc strains collected from Florida groves between 1997 and 2016. Our analysis revealed two distinct clusters of Xcc. Our data strongly indicate that the claimed eradication of the 1986 Xcc introduction was not successful and Xcc strains from 1986 introduction were present in samples from at least 8 counties collected after 1994. Importantly, our data revealed that the Cluster 2 strains, which are present in all 20 citrus-producing counties sampled in Florida, originated from the Xcc introduction event in the Miami area in 1995. Our data suggest that Polk County is the epicenter of the dispersal of Cluster 2 Xcc strains, which is consistent with the fact that three major hurricanes passed through Polk County in 2004. As copper-based products have been extensively used to control citrus canker, we also investigated whether Xcc strains have developed resistance to copper. Notably, none of the 343 strains contained known copper resistance genes. Twenty randomly selected Xcc strains displayed sensitivity to copper. Overall, this study provides valuable insights into the introduction, eradication, spread, and copper resistance of Xcc in Florida.

## Author summary

Sequencing and analyzing the genomes of 343 Xcc strains collected from 1997 to 2016 have enabled us to gain a deeper understanding of its introduction, spread, and eradication. This information is critical for optimizing strategies for responding to the invasion of quarantine pathogens. We have conclusively demonstrated that the Xcc introduction in 1986 was not successfully eradicated, three major hurricanes in 2004 play a critical role in spreading Xcc throughout Florida, and Polk County is the epicenter of Xcc dispersal. Additionally, none of the tested Xcc strains displayed resistance to copper, consistent with the absence of copper resistance genes in their genome.

## Introduction

Citrus canker, caused by *Xanthomonas citri* pv. citri (Xcc), ranks among the most important plant diseases globally. Xcc infects leaves, fruit and stems, giving rise to canker lesions, twig dieback, leaf and fruit drop, as well as tree decline, resulting in reduced yield, and quality [1]. Xcc infects plants through wounds or natural openings such as stomata [2]. Originally from Asia, Xcc has spread to most citrus-producing countries including the United States and Brazil [3,4]. Xcc comprises multiple pathotypes, including A, A*, and A^w^. Xcc A, the most prevalent worldwide, can infect all commercial citrus cultivars. In contrast, Xcc A^w^ and Xcc A* have a restricted host range, primarily affecting Mexican lime (Citrus aurantifolia) and alemow (*Citrus macrophylla*) [2,5,6]. In addition to Xcc, another pathovar, *Xanthomonas citri* pv. aurantifolii (Xca) also causes citrus canker disease. Xca induces canker B and canker C on limited hosts and these strains have only been reported in South America [7]. Due to its significant impact on the citrus industry, quarantine measures have been implemented in canker-free citrus-producing regions such as countries in the Mediterranean region and Australia. Although citrus canker was reported in Australia and South Africa, it was successfully eradicated [8]. In canker-endemic regions, it is often managed through foliar sprays of antimicrobials, such as copper products or antibiotics, windbreaks, disease-free nursery plants, and avoiding overhead irrigation [9].

Citrus canker was initially reported in Florida, USA in 1910, stemming from imported seedlings from Japan, and it was declared to be successfully eradicated in 1933 [7]. However, in 1986, citrus canker was again found in Manatee County, Florida and was officially declared eradicated by 1994 [2]. This declaration has since been questioned [10]. A third introduction of citrus canker was reported in the Miami area in 1995 [2]. Despite extensive eradication efforts by state and federal agencies (Florida Department of Agriculture and Consumer Services (FDACS), the Division of Plant Industry (DPI), and the USDA Animal and Plant Health Inspection Service (APHIS)), complete eradication of citrus canker introduced in 1995 in Florida proved to be unattainable. The mandatory eradication program for citrus canker in Florida was terminated in 2006 due to several factors. By 2006; citrus canker had spread throughout the state; depleting the funds allocated for eradication. Furthermore, numerous lawsuits were filed against the eradication program by Florida residents. Since then, the Florida citrus industry has adopted an integrated citrus management approach including copper applications to control canker [1]. Additionally, streptomycin and tetracycline have been used via foliar sprays on citrus to control canker or Huanglongbing in Florida since 2012 and 2016, respectively [11,12].

The primary objectives of this study are to address the following questions: whether Xcc strains introduced in 1986 were completely eradicated, how Xcc strains spread to various locations in Florida, and whether Xcc strains possess copper resistance genes. To tackle these questions, we employ phylogenomic analysis [13]. The sequencing of the Xcc genome was first accomplished in 2002 [14], marking one of the first sequenced plant pathogens. Since then, many more Xcc genomes and related strains have been sequenced [15–28]. In this study, we conducted genome sequencing of 343 Xcc strains collected from Florida citrus groves between 1997 and 2016. Our genomic analysis has furnished valuable insights into Xcc eradication, dispersal, and copper resistance.

## Results

### Genome sequencing of 343 Xcc strains collected from 1997 to 2016 in Florida

**Fig 1:**
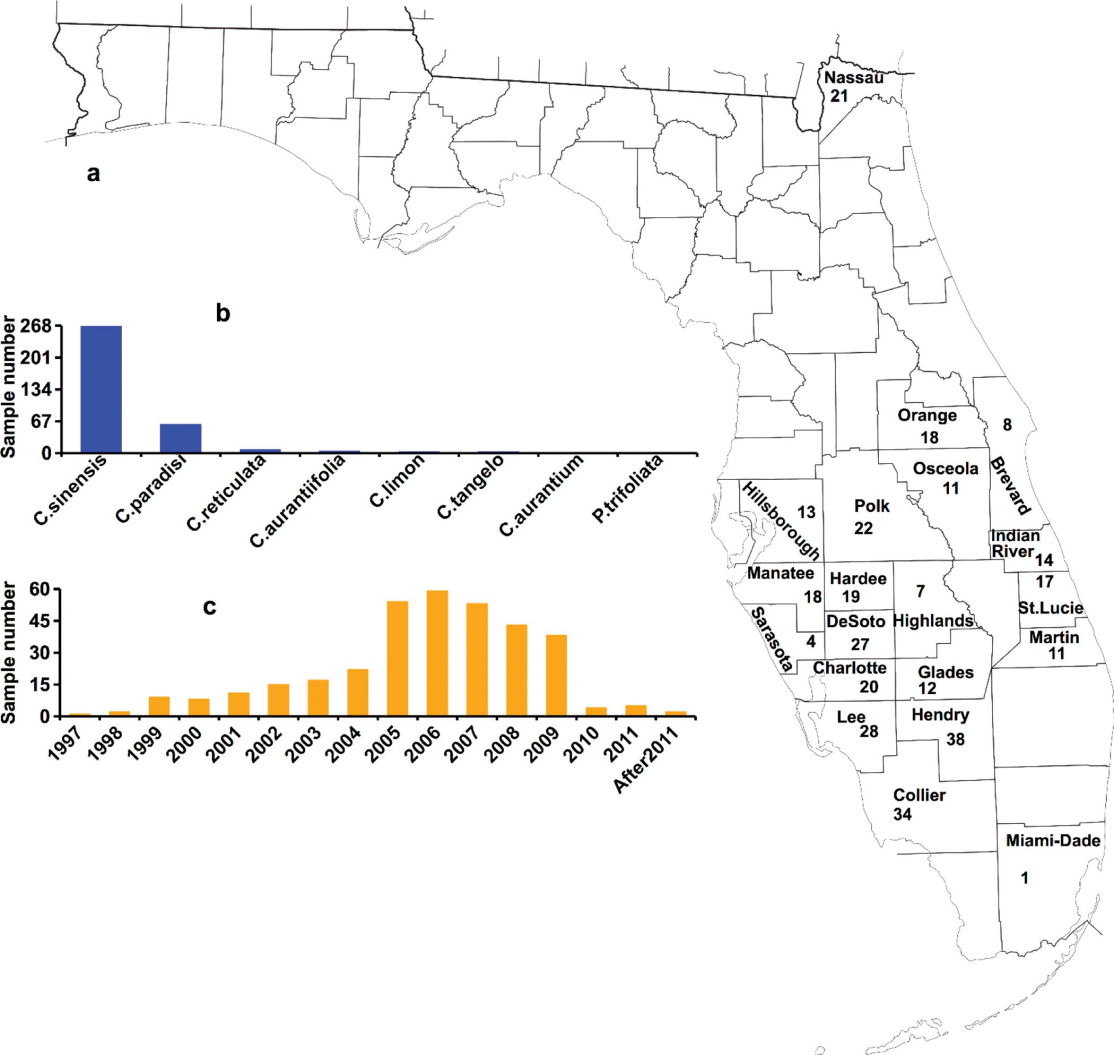
Sample information of 343 Xcc A strains from Florida. a, Geographic distribution of sampling sites. b, Host distribution of 343 samples. c, Time distribution of 343 samples. Base map of Fig 1A was generated from the website of the public domain U.S. Census Bureau (https://www2.census.gov/geo/maps/general_ref/stco_outline/cen2k_pgsz/stco_FL.pdf).

**Fig 2:**
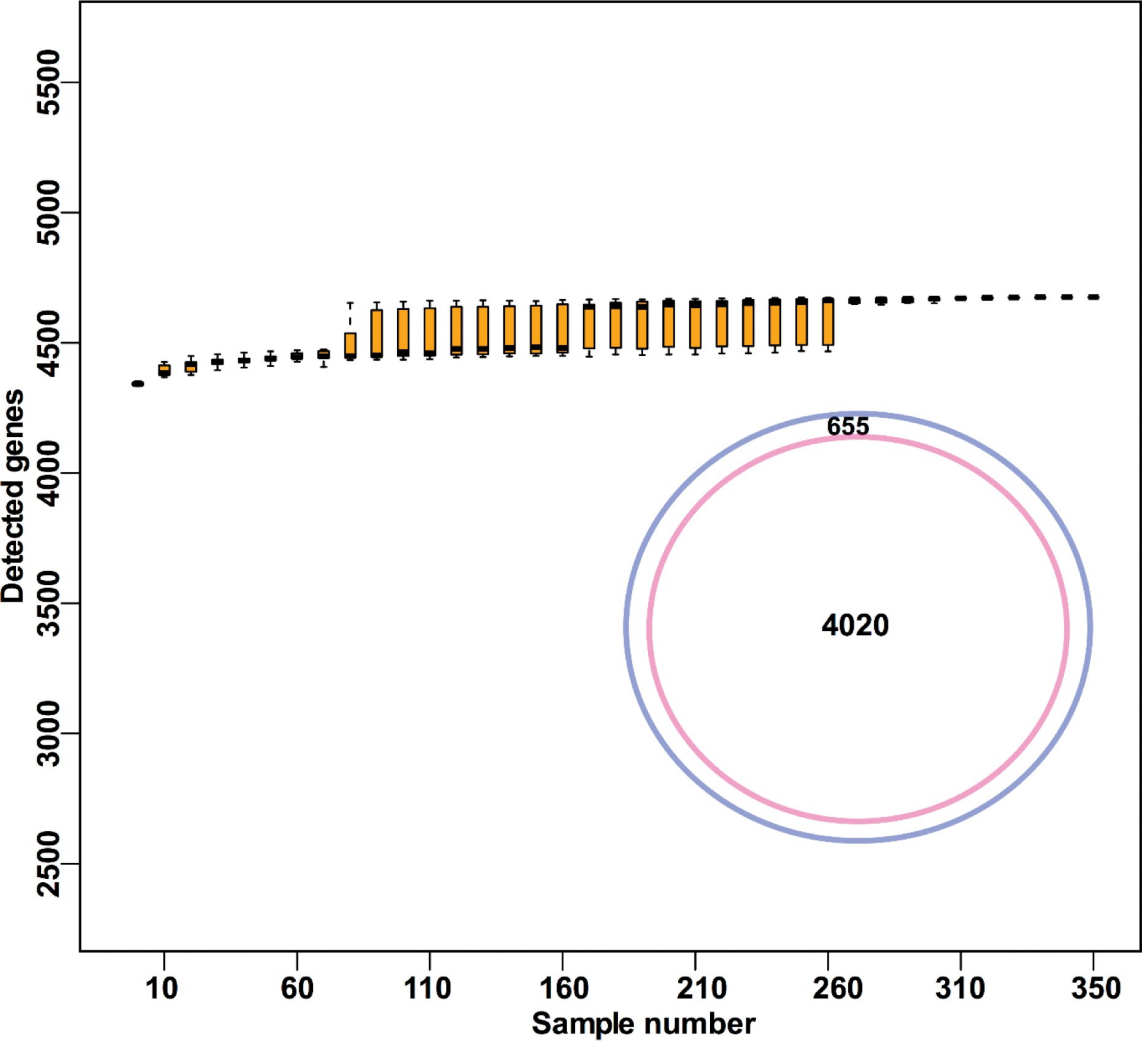
The pan genome of 351 Xcc A strains. a, Rarefaction curve of detected genes in pan genome based on 100-fold permuted sampling orders, center value represents the median of detected genes. b, The number of pan and core genomes of 351 Xcc A strains.

In total, we sequenced the genomes of 343 Xcc strains collected over 20 years (from 1997 to 2016) from various citrus groves in 20 Florida counties (Fig 1 and Tab A in S1 Dataset). The average sequencing coverage depth was 272.2 × based on BGISEQ short read sequencing. De novo assembled genome was obtained for each strain with an average of 4 scaffolds (range: 3 to 20), and a genome size of 5.21 Mb (range: 5.18 Mb to 5.38 Mb). The average of genome completeness for each strain was 98.3% (range: 97.43% to 98.44%) and 100% (range: 99.64% to 100%) as estimated by comparing with reference genome of Xcc 306 and single copy marker genes, respectively (Tab B in S1 Dataset). This result suggests that the assembled genomes meet the minimum information about a genome sequence (MIGS) specification [29]. On average, 4445 genes were predicted for each genome (Tab B in S1 Dataset). The average nucleotide identity (ANI) values among the 343 newly sequenced strains and Xcc 306 were 99.96–99.99% (Tab C in S1 Dataset). The ANI values among 343 newly sequenced strains and pathotypes Xcc A^w^ or Xcc A* ranged from 99.54% to 99.56% and 99.61% to 99.64%, respectively (Tab C in S1 Dataset). The 343 Xcc strains did not contain *avrGf1* (syn. *xopAG*), which is present in Xcc A^w^ and *X*. *vesicatoria* LMG911 [30], *xopC1*, which is present in Xcc A*, and *xopAF*, which is present in Xcc A^w^ and Xcc A*, but not in Xcc A [23]. These results suggest that the 343 Xcc strains belong to pathotype A. These strains exhibited a high degree of conservation in genomic sequence despite being isolated from different citrus cultivars, at different times, and in different locations (Fig 2 and Tab A in S1 Dataset).

### Two clusters of Xcc strains were identified among Florida isolates

Using the genomes of the 343 newly sequenced strains obtained through BGISEQ short-read sequencing, along with the seven previously reported Xcc strains from Florida [23], and the reference strain Xcc 306, we inferred the pan-genome of Xcc strains (Fig 2). The pan-genome comprised 4,020 core genes (present in all genomes) and 655 accessory genes (Fig 2A). Among these accessory genes, 216 were present in only one strain, and 177 were unique to the reference strain Xcc 306 (Tab D in S1 Dataset). A rarefaction analysis of the pan-genome, along with heap’s law estimation (alpha value = 2.0), indicted that the gene content for Xcc strains reached a plateau (Fig 2B), further supporting the nature of highly conserved genome sequences among Xcc A strains.

The mean ratio of the recombination rate to the mutation rate (ρ/θ), as estimated by ClonalFrameML, was 0.0264 (SD: 0.0046); the average length of recombined fragments (δ) was 98.3 bp (SD: 18.6 bp); and the average relative contribution of recombination and mutation (r/m) was 0.286 (SD: 0.0005). These values suggest that the Xcc population in Florida was effectively clonal in structure (S1 Table). Tests of selection, using the FUBAR (Fast Unconstrained Bayesian Approximation) method, identified 20 genes that underwent positive selection (S2 Table). By mapping the raw reads against the reference genome of Xcc 306, we identified 2,146 mutations, including single nucleotide polymorphisms (SNPs), insertions, and deletions (INDELs), for the 343 newly sequenced Xcc strains. These mutations were primarily concentrated in the plasmids and non-coding regions (S3 Table). Based on the variation participation analysis (VPA) using profile of genomic mutation and gene presence/absence (S4 Table), we found that both time and geographic location significantly contributed to the genomic variation observed among Xcc A strains in Florida.

**Fig 3:**
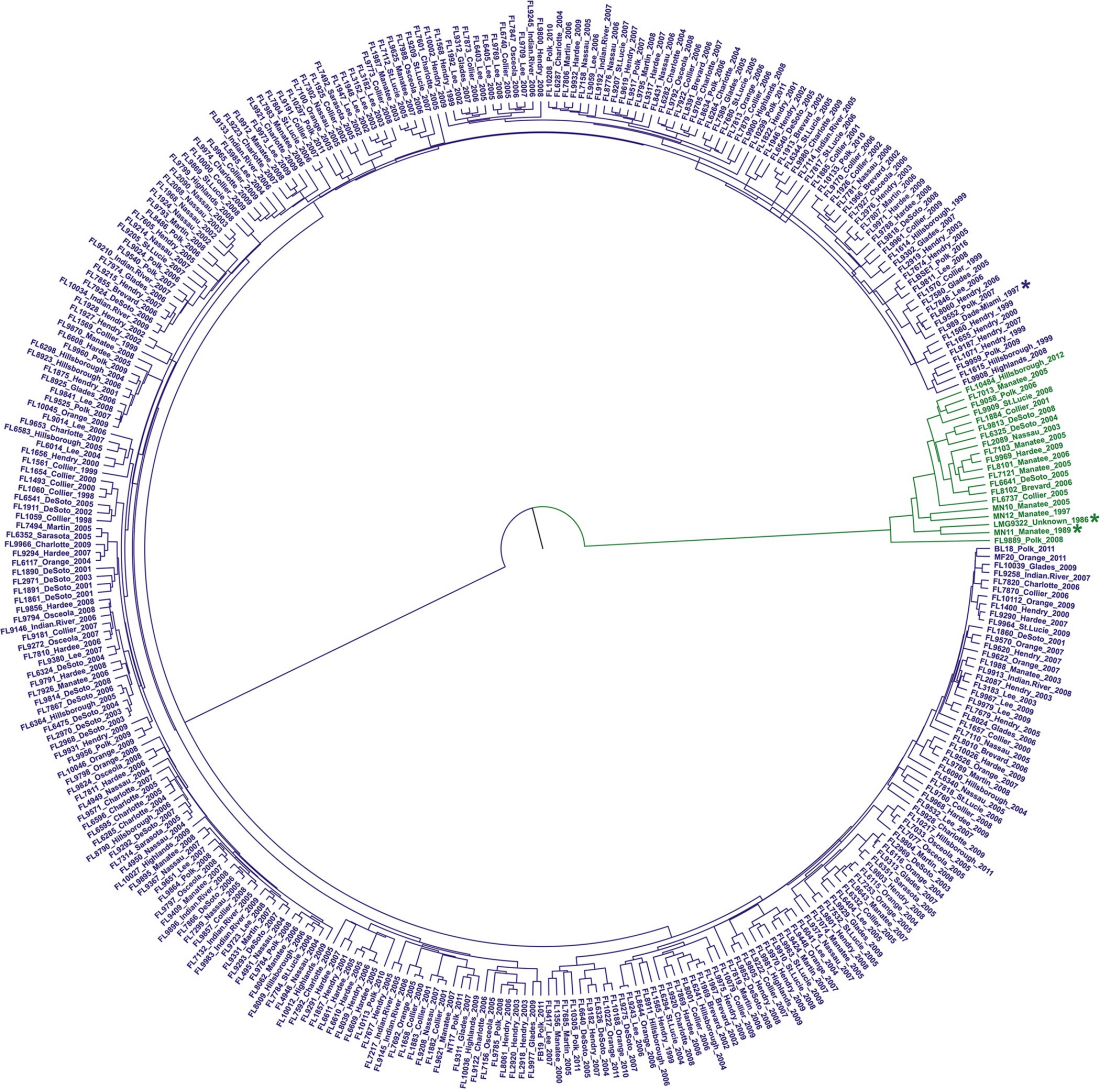
Population structure of Xcc A Florida strains based on phylogenetic relationship. The phylogenetic tree was performed using BEAST program based on SNP sequences of 351 Xcc A strains from Florida. *, represented the strain associated the Xcc introduction events in Florida. Green and purple indicate Cluster 1 and Cluster 2, respectively.

**Fig 4:**
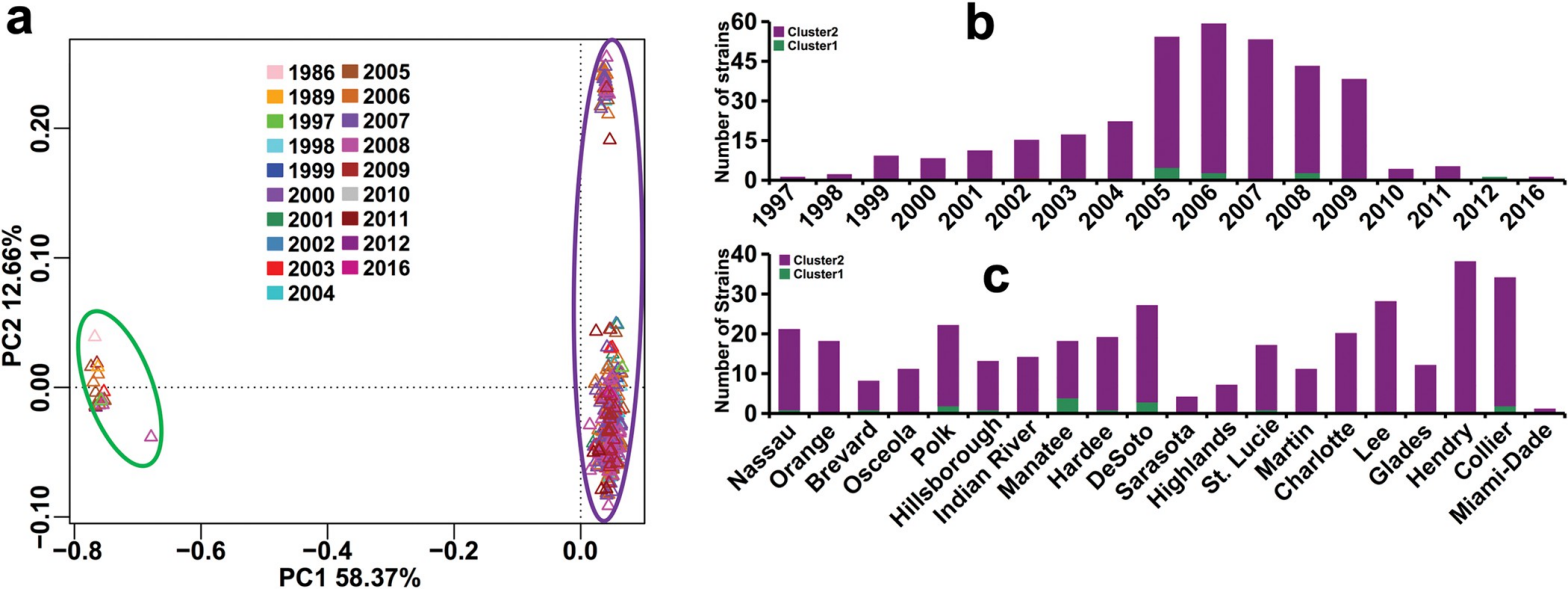
Population structure of Xcc A Florida strains using PCoA method. PCoA was performed based on the Bray distance among samples using genomic SNP mutation profiles of 351 Xcc A strains from Florida (a). b, Time distribution of two clusters of Xcc A strains. c, Geographic distribution of two clusters of Xcc A strains. Green and purple ellipses represent Cluster 1 and Cluster 2 respectively.

**Fig 5:**
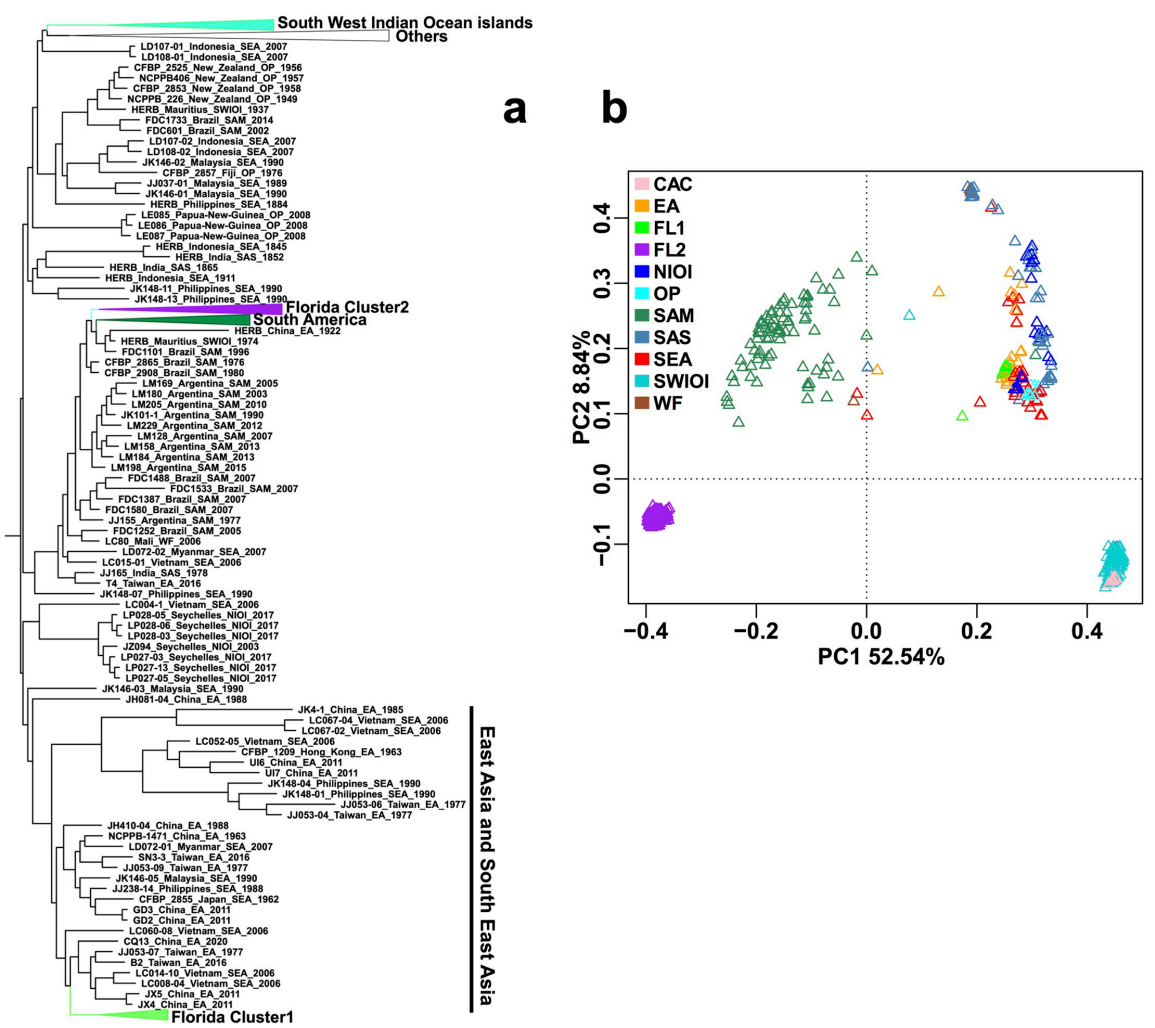
Population structure of Xcc A strains across globe. The phylogenetic tree was performed using FastTree program based on SNP sequences of 782 Xcc A strains (a). PCoA was performed based on the Bray distance among samples using genomics SNP mutation profiles of 782 Xcc A strains (b). CAC, Central America Caribbean; EA, East Asia; FL1, Florida Cluster 1; FL2, Florida Cluster 2; NIOI, North Indian Ocean islands; OP, Oceania Pacific; SAM, South America; SAS, South Asia; SEA, South East Asia; SWIOI, South West Indian Ocean islands; WF, West Africa.

To gain a better understanding of the evolution of Xcc A strains in Florida and their relationships with Xcc introduction events in the state, we employed both phylogenetic tree analysis and unsupervised machine learning methods such as Principal Coordinate Analysis (PCoA) to infer the population structure. In addition to the newly sequenced 343 strains, 10 publicly available genomes of Xcc from Florida were also included for analyses (Tab A in S1 Dataset). Among these 10 publicly available Florida Xcc genomes, two originated from Xcc strains introduced in Manatee in 1986, namely LMG9322 (collected in 1986) and MN11 (collected in 1989). In our newly sequenced 343 strains, there is one strain collected from the Miami area in 1997, which is close to the Xcc strains introduced in Miami in 1995 [10]. Unfortunately, there were no available genomes of Xcc associated with the first Xcc introduction event in Florida in 1910. Phylogenetic analysis, based on sequences of SNPs (351 strains) and single-copy core genes (353 strains), both indicated that Xcc A strains from Florida clustered into two groups (Figs 3 and S1). PCoA analysis based on the profile of genomic SNP mutations (351 strains), also supported the classification into two groups (Fig 4). Cluster 1 and Cluster 2 comprised 21 and 332 strains, respectively (Tab A S1 Dataset). Cluster 2 (containing strains collected from 1997 to 2016) included strains related to the Xcc introduction event in Miami in 1995 [10], exemplified by FL989 collected from Miami in 1997. In contrast, Cluster 1 (strains from1986 to 2012) formed a single clade genetically distant from the Miami strain. This cluster also encompassed strains related to the Xcc introduction event in Manatee in 1986, such as LMG9322 (collected in 1986) and MN11 (collected in 1989) (Figs 3, 4A, 4B and S1). Most strains in Cluster 1 were from Manatee, DeSoto, Polk, and Collier counties, whereas Cluster 2 strains were found in all 20 sampled counties (Fig 4C). Furthermore, we collected 431 publicly available genomes of Xcc A strains from around the world (Tab E in S1 Dataset). On a global scale, both phylogenetic and PCoA analyses based on genomic SNP mutations also indicated that Xcc A strains from Florida clustered into two groups (Fig 5). Strains from Cluster 1 were closely related to strains from East and Southeast Asia, whereas strains from Cluster 2 clustered together with strains from South America (Fig 5).

**Fig 6:**
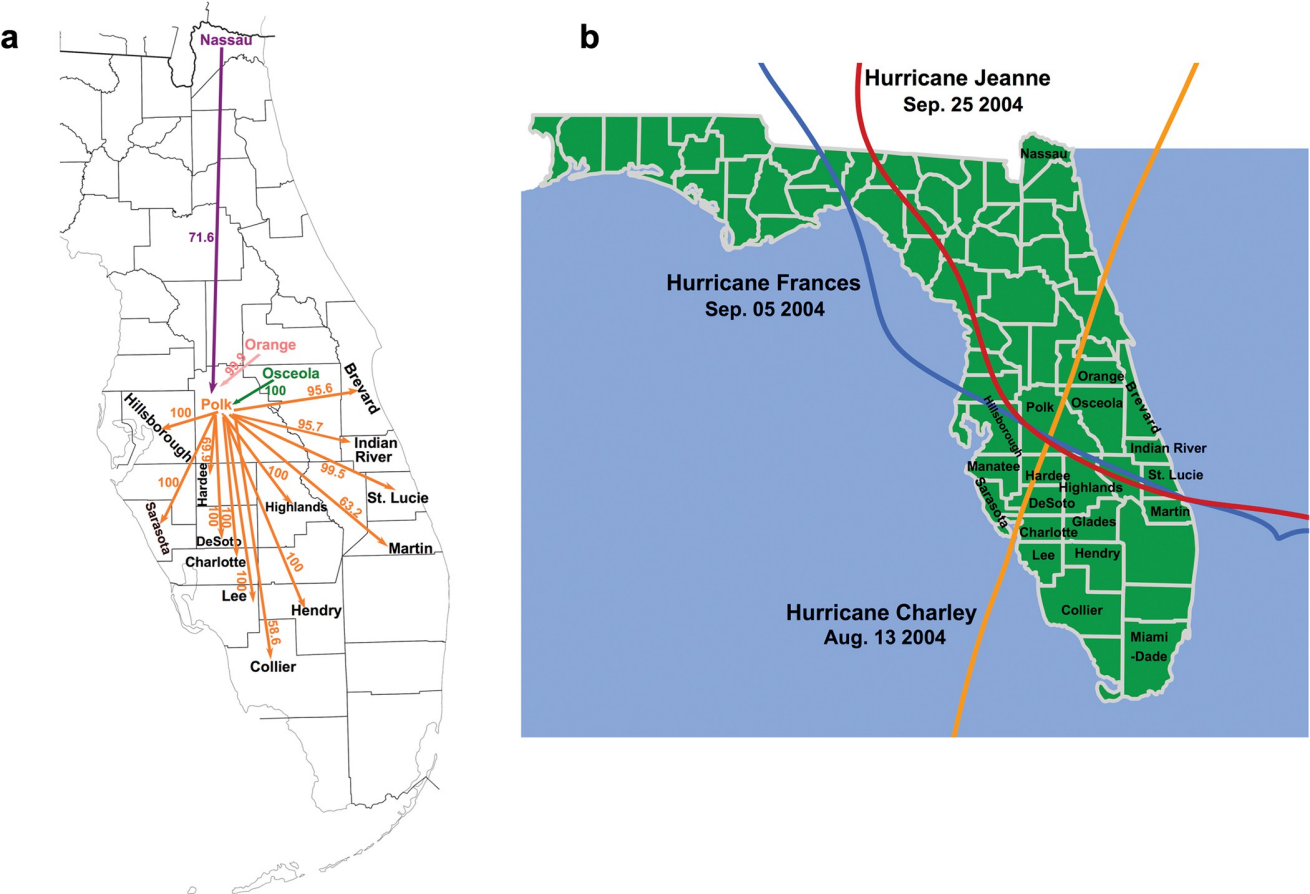
The movement of Xcc strains in cluster 2 across Florida. a, Determinants of geographical dispersal of Xcc in Florida. Discrete phylogenetic diffusion analyses were performed under an asymmetric diffusion model using Markov chain Monte Carlo (MCMC) implemented in BEAST v. 1.10. Bayesian stochastic search variable selection (BSSVS) was used to determine the significant pathways of spatial diffusion using spreaD3 v0.9.6. The number aside the line was the value of posterior probability. Arrows showed the direction of transmission between location. Color of arrows showed the resource location of Xcc transmission. b, The paths of three major hurricanes in Florida in 2004. Base map of Fig 6 was generated from the website of the public domain U.S. Census Bureau (https://www2.census.gov/geo/maps/general_ref/stco_outline/cen2k_pgsz/stco_FL.pdf).

There was a significant time signal (i.e., an increase in genetic variation over time) in the phylogenetic relationships of Florida Xcc strains (R^2^ = 0.19, P value = 0.036). Therefore, we employed Bayesian analysis to reconstruct the geographical dispersal of Xcc in Florida. However, due to the small sample size of Cluster1, the signal was relatively weak and indicated few dispersal events for Cluster1. In contrast, for Cluster 2, Xcc strains were observed to spread from the east and northeast (Nassau, Orange, and Osceola County) to central Florida (Polk County), and from there, they spread in multiple directions. These multiple dispersal events of Cluster 2 suggested that Polk County served as the central point for Xcc dispersal (Fig 6A). This observation aligns with the fact that in 2004, three major hurricanes passed through Polk County and its neighboring counties (Fig 6B). Notably, approximately 81.6% of the Cluster 2 strains were collected in or after 2004.

### Mutations in genes involved in pathogenicity and fitness among the two clusters of Xcc strains

The gene contents of the two Xcc clusters were similar, with a few unique genes identified for each cluster. Furthermore, each cluster displayed numerous unique genomic mutations, including SNPs and INDELs (S2 Fig). We conducted a detailed assessment of mutations in pathogenicity and fitness-related genes in the 343 strains. Genes containing SNPs and INDELs were notably overrepresented in categories such as the two-component system, glycolysis/gluconeogenesis, pyruvate metabolism, quorum sensing, and protein kinases (Fisher’s exact test, p-value < 0.05). However, this overrepresentation was not observed in known pathogenicity genes such as type III secretion system and effectors (Fisher’s exact test, p-value > 0.05). Mutations in the coding regions were also identified in genes encoding proteins related to bacterial motility, peptidases, chemotaxis, transcription factors, ABC transporters, and bacterial secretion systems (S5 Table). Additionally, genes associated with pathogenicity and fitness, including those involved in chemotaxis, T3SS and effectors, T2SS, T4SS, T6SS, T4 pilus, flagella, rpf regulator, iron metabolism, polysaccharide utilization enzymes, biofilm formation, and kinases, contained non-synonymous SNPs or INDELs (Tab F in S1 Dataset). It is noteworthy that none of these SNPs or INDELs were universally present in all 343 strains, suggesting the randomness of mutations.

### Copper resistance

Copper-based antibacterial products have been extensively used in Florida citrus groves since the citrus canker eradication program ended in 2006. We first analyzed copper resistance genes. *copL*, *copA*, *copB*, *copM*, *copG*, *copC*, *copD*, and *copF* genes, which have been reported to be responsible for copper resistance. Among them, *copL*, *copA*, and *copB* are recognized as primary contributors (S6 Table) [31]. Notably, none of these copper resistance genes were identified in the 343 Xcc strains. Subsequently, we conducted tests on 20 randomly selected Florida Xcc isolates to assess their sensitivities to copper (S7 Table). The 20 tested Florida Xcc strains exhibited a sensitivity to copper similar to that of Xcc306, with a minimum inhibitory concentration (MIC) of CuSO_4_ at 100 μg/mL (S7 Table). This finding suggests that Xcc strains have not developed resistance to copper based antimicrobials at least via well-known resistance genes.

## Discussion

In this study, we classified the Xcc population in Florida, US into two clusters, suggesting two distinct Xcc introduction events. Xcc has been reported to be introduced into Florida three times: first in 1910 near the Florida-Georgia border, then in 1986 in the Tampa Bay area, and finally in 1995 in the Miami area [2]. The first two introductions were declared eradicated in 1933 and 1994, respectively [2]. Due to the lack of genome sequences for the first Xcc introduction event in 1910, we cannot determine whether any Xcc strains were associated with this introduction event. However, the eradication of the Xcc introduction in 1986 was questioned because Xcc was discovered in the same region three years later [2,10]. Our study verifies this speculation with 18 samples collected after 1994 when the eradication was claimed to be completed. The Xcc strains identified in 1986 likely belong to Cluster 1 as indicated by the presence of two strains (MN11 and LMG9322) isolated near 1986 and their primary distributions in Manatee, De Soto, and Polk, adjacent to the Tampa Bay area. Cluster 2 includes the Miami strain (FL989) collected in 1997, suggesting it resulted from the Xcc introduction in 1995 in the Miami area. A previous study suggested that the Xcc introduction in 1995 in the Miami area initially spread to North or Northeast Florida. It was reported that during a rainstorm in January 1996, a tornado passed through the citrus canker-infected area of Miami on a Southwest to Northeast track [32]. In line with this, our findings indicate that Cluster 2 strains spread from North or Northeast to Central Florida and beyond. Polk County was suggested to be the dispersal center of Cluster 2 strains, likely spread by the three major hurricanes in 2004 that passed through Polk County. Most sampling points for Cluster 2 strains in our study were in or after 2004. In this study, we lacked the genomes of Xcc from a few citrus-producing counties that were and may still be infected, such as Palm Beach, and Broward counties. Importantly, Cluster 1 strains were closely related to strains from East and Southeast Asia, while Cluster 2 strains were closely related to strains from South America, suggesting the possible origins for those two Xcc introduction events in Florida. In accordance with this potential Xcc spreading path from South America, citrus canker was first reported in Brazil in 1957 and Argentina in 1974 [33].

Eradication has been successfully used for the management of multiple invading plant diseases. For instance, citrus canker has been successfully eradicated three times in Australia following introductions in 1991, 2004, and 2018 [34]. However, in Florida, only one of the three Xcc eradication campaigns was successful whereas the campaigns for the 1986 and 1995 Xcc introductions failed. One potential reason for this could be hurricanes in Florida, which extensively spread Xcc in 2004, as analyzed in our study and elsewhere [32,35]. It’s noteworthy that at least 6 hurricanes passed by Florida between 1986 and 1994, including Hurricane Andrew in 1992, which might have contributed to the distribution of Xcc introduced in 1986. Thus, how to effectively eradicate invading pathogens in locations like Florida needs to be investigated.

Copper-resistant Xcc strains have been reported in Argentina [31] and on the French islands of Réunion [36] and Martinique [37], but such resistance has not been reported in Florida. Despite the frequent use of copper products in Florida citrus groves since 2006, at this stage, we found no evidence in our collection of Xcc strains that they have evolved resistance to copper, at least not via well-known resistance genes. The majority of copper resistance genes in plant-pathogenic bacteria are known to be plasmid-borne including copper-resistant strains of *X*. *citri* pv. *citri* A44 and *X*. *euvesicatoria* pv. *citrumelonis* 1381 [38]. Consequently, horizontal transfer of copper resistance genes by conjugation is the primary mechanism for the acquisition of copper resistance by bacteria. In Florida, multiple copper-resistant bacteria have been identified including *X*. *euvesicatoria* pv. *citrumelonis* 1381, *X*. *euvesicatoria* pv. *euvesicatoria*, *X*. *euvesicatoria* pv. *perforans* [38] and a citrus epiphytic strain of *Stenotrophomonas maltophilia* [39]. Many copper-resistant bacteria do not occur in the same hosts or the same locations as Xcc in Florida. For instance, *X*. *euvesicatoria* pv. *citrumelonis* 1381 causes citrus bacterial spot specifically in nursery trees, while *X*. *euvesicatoria* pv. *euvesicatoria* and *X*. *euvesicatoria* pv. *perforans* infect peppers or tomatoes, but not citrus. The susceptibility of Xcc strains to copper in Florida suggests that copper-based products remain a viable solution for controlling citrus canker [1]. On the other hand, identification of copper-resistant citrus epiphytic bacteria in Florida indicates the need to closely monitor copper resistance development in Xcc and develop other efficient and sustainable control approaches, such as generating canker-resistant citrus cultivars by mutating the canker susceptibility gene *LOB1* [40–45].

In sum, we have conducted genome sequencing of 343 Xcc strains collected from 1997 to 2016. We have demonstrated that the Xcc population in Florida can be classified into two clusters, with Cluster 1 strains introduced in 1986 and Cluster 2 strains introduced in 1995. We have shown that the Xcc introduction in 1986 was not successfully eradicated and the three hurricanes in 2004 played a crucial role in spreading Xcc in Florida with Polk County being the center of Xcc dispersal.

## Materials and methods

### DNA sequencing and genome assembly

Glycerol stocks of Xcc strains isolated from citrus leaves with canker symptoms collected previously in Florida citrus groves by DPI were stored in a -80°C freezer. These isolates were plated on nutrient agar medium and streaked three times to obtain pure colonies. Genomic DNA extraction for each strain was performed using the Wizard Genomic DNA Purification Kit (Promega, Madison, WI) according to the manufacturer’s instructions. DNA quality and quantity were determined using the Nanodrop One Microvolume UV-Vis Spectrophotometer (Thermo-Fisher Scientific, Waltham, MA) and electrophoresis on a 0.8% agarose gel. The DNA samples were then stored at -80°C until further use.

Shotgun genomic library preparation and sequencing of 343 samples (Tab A in S1 Dataset) were performed per the manufacturer’s protocol using the BGISEQ500 platform at BGI-Shenzhen, China as previously described [46]. Briefly, 500 ng of input DNA was used for library generation and fragmented ultrasonically to yield 400 to 600 base pairs (bp) of fragments. DNA fragments were then end-repaired and A-tailed, and adaptors with specific barcodes were added. PCR amplification of DNA fragments was carried out to generate a single-strand circular DNA library. The DNA libraries were sequenced by BGISEQ500 using a paired-end 100-bp sequencing strategy. On average, more than 1,435 million bp (Mb) of raw data were generated for each strain (Tab B in S1 Dataset).

The raw reads obtained from BGISEQ50 sequencing were used to generate clean reads by removing adaptor sequences, trimming, and removing low-quality reads (reads with N bases and a minimum quality threshold of 20) at BGI-Shenzhen, China. Clean reads were further trimmed using Sickle software [47] and trimmed reads < 50 bp were discarded. De novo genomic assembly was performed using SPAdes version 3.13.0 with default parameters [48]. The assembled genomes from SPAdes were further scaffolded based on the reference genome of Xcc 306 using MEDUSA version 1.6 with default parameters [49]. The genome quality was assessed using QUAST version 2.3 [50] compared with reference genome of Xcc 306 and CheckM program version 1.1.2 using single copy marker genes [51]. Gene prediction of each genome was performed using Prokka automatic pipeline version 1.14.6 [52]. The summary of genome sequencing, assembly, and gene prediction for the 343 Xcc A strains is provided in Tab B in S1 Dataset.

### Comparative genomic analysis

We performed a comparative genomic analysis of 351 Xcc strains (Tab A in S1 Dataset), including the 343 newly sequenced strains from BGISEQ in this study, 7 strains from a previous study [23], and the reference strain Xcc 306. The average nucleotide identity (ANI) values between genomes were calculated using pair-wise blast alignment. Based on the genes predicted by Prokka, the pan-genome and core genomes of Xcc were constructed using the Roary program version 3.12 [53] with parameters set to an identity of 90%. The rarefaction of detected genes in the pan genome based on 100-fold permuted sampling orders was performed as described elsewhere [54]. The heaps law estimate of the pan-genome was performed using the micropan package version 2.1 [55] in R program version 4.2. To calculate the ratio of recombination rate to mutation rate (ρ/θ) and the relative contribution of recombination and mutation (r/m), we performed recombination analysis using ClonalFrameML program version 1.11 [56] based on a total of 3,776 single-copy core genes. The nucleotide sequences of core genes were aligned using MUSCLE version 3.8.31 [57] and poorly aligned regions were removed using trimAl v1.2 [58], and then the maximum likelihood phylogenetic tree was constructed using FastTree Version 2.1.7 [59]. The concatenated alignment and ML phylogenetic tree of the core genome generated by FastTree were used as inputs for ClonalFrameML. To assess the contribution of positive selection on the Xcc strains, the analysis of Unconstrained Bayesian AppRoximation (FUBAR) [60] implemented in HYPHY 2.2 software was carried out with 4,021 shared genes among the genomes of Xcc strains. To verify the reliability of the analysis, the FUBAR analysis with 400 Grid points, five independent runs and 2,000,000 iterations were performed.

The SNPs and INDELs across the genomes of the 343 newly sequenced Florida Xcc strains and 439 publicly available Xcc strains (Tabs A and E in S1 Dataset) were identified by mapping the raw short reads to the reference genome of Xcc 306 using bowtie2 version 2.2.6 [61], and samtools version 1.2 [62]. Briefly, the raw reads were aligned against the reference genome of Xcc 306 using bowtie2 with default parameters. The alignment files, in BAM format, were used to call SNPs and INDELs using the mpileup pipeline and quality filtering with bcftools integrated in the samtools program. The annotation of SNPs and INDELs was obtained according to the gene annotation of the reference genome of Xcc 306. KEGG pathway enrichment analysis for genes containing SNPs and INDELs was performed using Fisher’s exact test. The variation participation analysis of time and geographic location with PERMANOVA analysis was carried out based on both the pan-gene presence and genomic mutation profiles using the VEGAN package in R software [63]. The population structure of Xcc strains was determined using both PCoA and phylogenetic relationship methods. PCoA analysis of Xcc strains was performed with Bray distance based on genomic SNP mutation profiles using the WGCNA package in R software [64]. The maximum likelihood phylogenetic trees based on SNP sequences and single copy core genes of Florida Xcc A strains was constructed using FastTree Version 2.1.7, and then examined the time signal using TempEst version 1.5.3 [65]. We further assessed the population structure and evolutionary dynamics of Florida Xcc A strains using the BEAST2 program version 2.6 [66] based on SNP sequences. The maximum clade credibility tree was generated using TreeAnnotator v2.6.6 [66] and visualized in FigTree v1.4.4 [66]. Discrete phylogenetic diffusion analyses were performed under an asymmetric diffusion model using Markov chain Monte Carlo (MCMC) implemented in BEAST v. 1.10 [67]. Bayesian stochastic search variable selection (BSSVS) was used to determine the pathways of spatial diffusion using spreaD3 v0.9.6 [68,69].

### Copper resistance gene identification

To identify potential copper resistance genes, we used both DNA and protein sequences of the 343 Florida Xcc A strains and Xcc 306 to align them with reference genes of copper resistance genes [31] using blastn and blastp programs (e-value less than 1e-5) from the NCBI blast tool [70].

### Determination of minimum inhibitory concentrations (MICs)

The MICs of copper (CuSO4) against 20 randomly selected Florida Xcc isolates were determined using the broth microdilution method [71]. Xcc 306 was included as a control for comparison. Briefly, the bacterium was grown to the exponential phase in nutrient broth (NB) at 28°C with shaking at 200 rpm for 6–8 h. The cultures were standardized to an OD600 of 0.03 (5 × 107 colony-forming-units [CFU]/mL) and then aliquoted into wells of a 96-well plate, 180 μL per well. The initial test concentrations of the compounds were diluted (1:10) in the culture (20 μL of compound added into 180 μL of bacterial culture) and incubated at 28°C under stationary conditions. The cultures were monitored at 24 and 48 h at OD600, and the lowest concentration resulting in no growth after 48 h compared with the control samples was defined as the MIC for Xcc. Bacterial suspension without the tested compound and NB medium without bacterial culture were used as positive and negative controls of bacterial growth, respectively. All determinations were conducted in eight replicate wells and repeated three times.

### Bacterial sensitivity to copper assays

Xcc strains were examined for sensitivity to copper through determination of the respective minimum inhibitory concentration (MIC) of CuSO_4_ against the bacterial strains, using a broth microdilution method [71]. Briefly, the bacterium was grown to the exponential phase in NB at 28°C with shaking at 200 rpm for 6–8 h. The cultures were standardized to an OD_600nm_ of 0.03 (5 × 10^7^ CFU/mL) in NB and aliquoted into wells of a 96-well plate, 180 μL per well. The initial test concentrations of the compounds were diluted (1:10) in the bacterial culture (20 μL of compound added into 180 μL of bacterial culture) and incubated at 28°C under stationary conditions for 48 h. Bacterial growth was measured at OD_600nm_, and the lowest concentration resulting in no growth compared with the control samples was considered the MIC for the bacterium. Bacterial suspension without the tested compound and NB medium without bacterial culture were used as positive and negative controls of bacterial growth, respectively. All assays were performed in 4 replicate wells and repeated three times.

## Supporting information

S1 Table The recombination rate of genomes of Florida XccA population. (XLSX)

S2 Table Genes from XccA strains under positive selection. (XLSX)

S3 Table The summary of genomic mutations of 343 XccA strains. (XLSX)

S4 Table Contribution of time and location on genomic variations. (XLSX)

S5 Table The KEGG pathway enrichment analysis of genes containing mutations in the 343 XccA strains. (XLSX)

S6 Table Analysis of copper resistance genes in 343 *Xanthomonas citri* subsp. citri strains isolated in Florida. (XLSX)

S7 Table Sensitivity of 20 Florida Xcc strains to copper as determined by minimum inhibitory concentration (MIC) assays. (XLSX)

S1 Fig The phylogenetic tree of 353 Florida Xcc A strains. The phylogenetic tree was performed using FastTree program based on core gene DNA sequences of 353 Florida Xcc A strains. *, represented the strain associated the Xcc introduction events in Florida. Green and purple indicate Cluster 1 and Cluster 2, respectively.
(TIF)

S2 Fig Venn plot depicting the number of genes (a) and genomic mutations (b) among the two clusters of Xcc A strain.
(TIF)

S1 Dataset A. The sample information of 343 newly sequenced and 10 publicly available genomes of Florida Xcc A strains used in this study. B. The summary of genome sequencing, assembly, gene prediction, and NCBI accession information of 343 XccA strains. C. The average nucleotide identity of genomes among 343 Florida XccA and reference strains. D. The core and accessory genes of pan genome of 350 Florida XccA strains. E. The sample information of 431 Xcc A strains worldwide from public database. F. The mutation information of genes involved in pathogenicity or fitness from the 343 XccA strains.
(XLSX)
