# Supplementary figures and images for: Phylogenomic analysis of 343 Xanthomonas citri pv. citri strains unravels introduction history and dispersal paths
Source: PLoS Pathog. 2023 Dec 15;19(12):e1011876. doi: 10.1371/journal.ppat.1011876 (PMC10756548; doi:10.1371/journal.ppat.1011876)

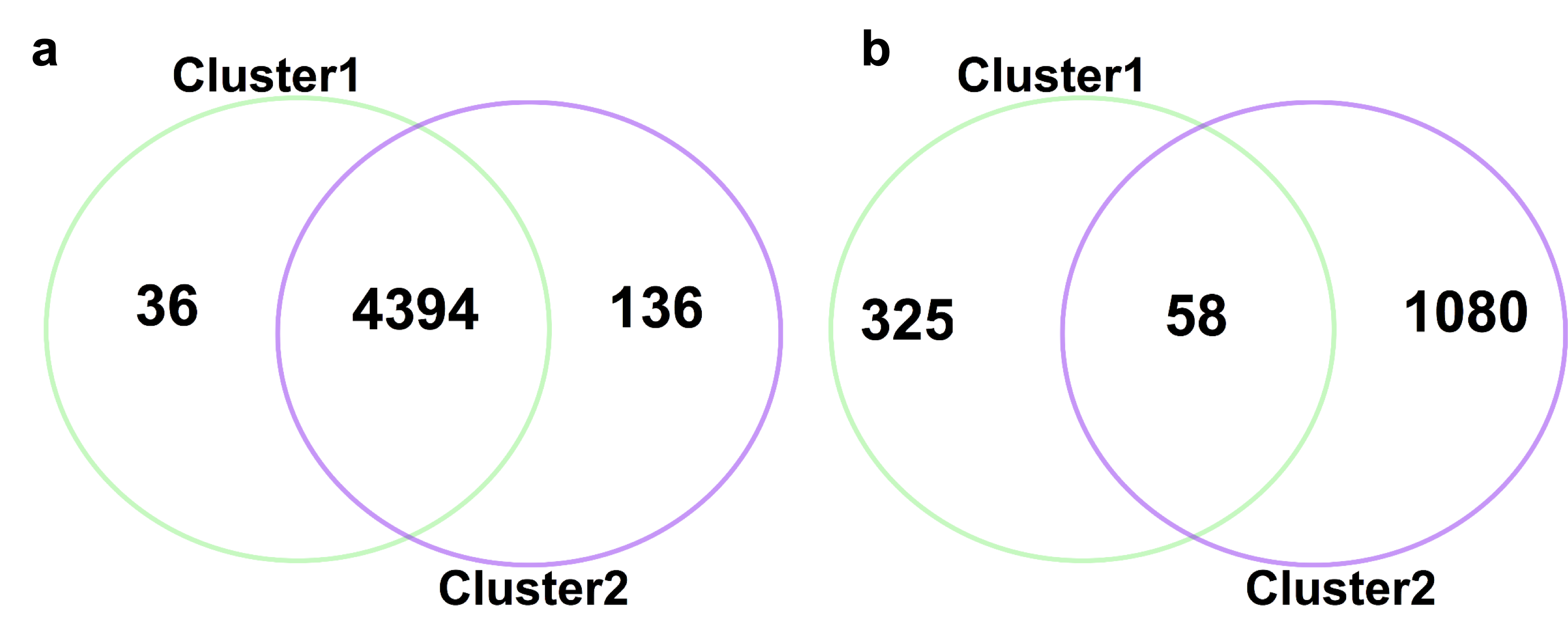

Supplement: S2 Fig — Venn plot depicting the number of genes (a) and genomic mutations (b) among the two clusters of Xcc A strain. (TIF) [file ppat.1011876.s009.tif]
